# Impact of near‐peer teaching on medical English learning motivation among medical students and residents

**DOI:** 10.1002/jgf2.629

**Published:** 2023-05-30

**Authors:** Yoji Hoshina, Limeisa, Hodaka Takaiso, Hidenori Maki, Toru Yoshino, Kiyoshi Shikino

**Affiliations:** ^1^ Department of Neurology University of Utah Salt Lake City Utah USA; ^2^ Tokushima University Tokushima Japan; ^3^ Misawa Airbase Hospital Aomori Japan; ^4^ Department of General Surgery Saiseikai Kanagawa Prefecture Hospital Yokohama Japan; ^5^ Okinawa Naval Hospital Okinawa Japan; ^6^ Department of General Medicine Chiba University Hospital Chiba Japan

## Abstract

The study evaluated the effectiveness of near‐peer teaching in medical English education and found that it led to higher levels of motivation, curiosity, initiative, self‐efficacy, learning goals, and psychological safety among participants compared to traditional teacher‐centered approaches. The study also observed behavioral changes and active engagement in English learning among participants. We hope that our activity will inspire other educators to adopt similar approaches in their medical English classes and promote more effective language learning among health professionals.

To the Editor

Globalization increased the demand for medical English education among health professionals, emphasizing the need to motivate medical students and residents to learn medical English.^1^ Traditional medical English classes have emphasized basic medical terminology and reading and writing in English. Although this teacher‐centered approach provides medical knowledge to students, it can compromise their motivation.^2^

The United States Medical Licensing Examination Study Group of Tokushima, launched in 2016, addresses this issue by adopting near‐peer teaching as a learner‐centered approach. This group aimed to promote student motivation and curiosity; cultivate their initiative, self‐efficacy, and English‐learning goals in a safe environment; and nurture self‐regulated lifelong English learners. A comprehensive depiction of the group is provided in a separate publication.^2^ The group expanded to the point of being able to conduct a monthly activity with different topics since 2022. During each session, one or two volunteers presented medical English‐related topics, including clinical rotation experience abroad. When no one had a topic, YH, HT, YT, and HM, who were medical students in 2016, shared their experiences to prepare for working in the United States. This helped participants visualize future opportunities and develop an international posture, thereby encouraging them to connect to the international community by learning English.

A survey using a seven‐point Likert scale (1 = strongly disagree, 7 = strongly agree) evaluated motivation, curiosity, initiative, self‐efficacy, learning goals, and psychological safety. The survey was conducted in May 2022 to evaluate the teacher‐centered approach and in March 2023 to evaluate the near‐peer teaching approach. Behavioral changes were analyzed in the second survey.

Fifty‐three members (25 students and 28 residents; response rate: 70.7%) who attended at least one session responded to the questionnaire. The mean motivation, curiosity, initiative, self‐efficacy, learning goals, and psychological safety were higher in the near‐peer teaching group than those in the teacher‐centered group (5.8 ± 1.2 vs. 3.9 ± 1.7, 5.8 ± 1.2 vs. 4.1 ± 1.7, 5.6 ± 1.3 vs. 4.0 ± 1.8, 5.2 ± 1.4 vs. 3.1 ± 1.6, 5.4 ± 1.4 vs. 3.7 ± 1.6, and 5.7 ± 1.4 vs. 3.9 ± 1.7, respectively; *p* < 0.01; Mann–Whitney *U* test). Forty‐seven participants (88.7%) exhibited at least one behavioral change (Table 1).

**TABLE 1:** Number of students who started learning English after the sessions.

Behavioral modification	Number of students (%)
TOEIC/TOEFL	29 (54.7)
USMLE	29 (54.7)
English lessons (include in‐person and online modes)	21 (39.6)
Language study in a foreign country	6 (11.3)
Observership or externship in a foreign country	15 (28.3)
Volunteer work in a foreign country	2 (3.8)
Work in the US medical system^a^	7 (13.2)

Abbreviations: TOEIC, Test of English for International Communication; TOEFL, Test of English as a Foreign Language; USMLE, United States Medical Licensing Examination.

^a^ Includes residency training, United States Naval Hospital, and United States Airbase Hospital.

Near‐peer teaching, in which non‐professional teachers in a similar social group help each other learn and learn by teaching themselves, improves participants' performance in various medical curricula.^3^, ^4^ This approach enhances tutors' and tutees' intrinsic motivation, which is essential in continuing to study the subject.^5^ It also creates a comfortable and safe educational environment and provides role models.^5^ This approach enabled participants to actively engage in English learning (Table 1). Notably, seven graduates, including YH, HT, TY, and HM, have started their careers in the US medical system over the past 7 years, becoming lifelong English learners. Thus, teaching peers strengthens lifelong learning, and students who teach are highly motivated to prepare thoroughly for their teaching, which serves as a powerful drive for learning and meaningfully adds to teachers' knowledge.^5^

In conclusion, our approach succeeded in increasing students' motivation to learn medical English, although the improvement in their reading and writing skills is unknown. Despite some limitations, including a single‐center design, sampling bias (some of the group members, especially those who tutored, may have been already highly motivated), non‐validated scales, and a pre‐post study design, we hope that our activity will inspire other educators to adopt similar approaches in their medical English classes and promote more effective language learning among health professionals.

## CONFLICT OF INTEREST STATEMENT

The authors have stated explicitly that there are no conflicts of interest in connection with this article.
